# Levonorgestrel only emergency contraceptive use and risk of ectopic pregnancy in Eldoret Kenya: a case-control study

**DOI:** 10.11604/pamj.2018.31.214.17484

**Published:** 2018-11-29

**Authors:** Sahara Shurie, Edwin Were, Omenge Orang’o, Alfred Keter

**Affiliations:** 1 Department of Reproductive Health, College of Health Sciences Moi University, Eldoret, Kenya; 2 Department of Medicine, College of Health Sciences, Moi University, Eldoret, Kenya

**Keywords:** Ectopic pregnancy, extra-uterine pregnancy, postcoital contraception, Levonorgestrel

## Abstract

**Introduction:**

ectopic pregnancy is one of the causes of maternal morbidity and mortality in sub-Saharan Africa. Levonorgestrel (LNG) only emergency contraceptive pill is a well-established emergency contraceptive pill that is administered within 72 hours after unprotected intercourse. This study aimed at determining whether or not there is a significant association between levonorgestrel emergency contraceptive use and the occurrence of ectopic pregnancy.

**Methods:**

case-control (1:3) study among 79 women with ectopic pregnancy (cases) matched against 237 women without (controls) at Moi Teaching and Referral Hospital in Eldoret, Kenya; Sociodemographic and clinical data were collected using a questionnaire. Association between ectopic pregnancy and LNG-EC was assessed using Pearson chi-square test. The relationship between outcome and exposure (while adjusting for confounders) was assessed using logistic regression model.

**Results:**

The mean age was 27.15 years. Both cases and controls were similar by age (p = 0.990), educational level (p = 0.850), marital status (p = 0.559), employment status (p = 0.186) and parity (p = 0.999). Seventy-eight (24.7%) participants had a history of miscarriage. A higher proportion of the cases had history of using LNG-EC compared to the controls (32.9% vs. 7.2%, p < 0.001). The use of LNG-EC portended more than nine times increased odds of ectopic pregnancy compared to other contraceptive methods {OR = 9.34 (95% CI: 3.9 - 16.0)}.

**Conclusion:**

levonorgestrel only emergency contraceptive use was associated with ectopic pregnancy. One of the limitations of this study is that we could not control for all confounders of ectopic pregnancy.

## Introduction

Globally, emergency contraception pills (ECPs) are widely used by women after unprotected intercourse to prevent unwanted pregnancies [1]. In many countries including Kenya; most pharmacies stock Levonorgestrel-only pills for emergency contraception (LNG-EC) and sell them over-the-counter form [2-5]. LNG-EC can prevent unwanted pregnancies with an efficacy of 52-94% when used within 12 hours of unprotected intercourse [5, 6]. Like other contraceptive methods, LNG-EC reduces the chance of pregnancy whether intrauterine or ectopic pregnancy [6]. However, cases of ectopic pregnancy following LNG-EC failure have been reported recently by various researchers in different countries [1, 7-9]. Previous systematic reviews have drawn the conclusion that the incidence of ectopic pregnancy following LNG-EC failure was 1.6% and this did not exceed the incidence in general female population [10]. However, most studies on contraception failure and pregnancy as the primary endpoint; did not report whether pregnancies following LNG-EC failure were intrauterine or extra-uterine [11]. Ectopic pregnancy is one of the causes of maternal morbidity and mortality in sub-Saharan Africa [12]. The incidence of ectopic pregnancy in Western Kenya continues to be a major cause of admission in health facilities [13]. Studies on the association between LNG-EC pills and ectopic pregnancy have had conflicting results [14, 15]. According to the latest Kenya demographic health survey, the prevalence of contraceptive use among women in their reproductive age is increasing [13]. Increased use of emergency contraception among women is due to increase in demand for prevention of unplanned pregnancies [3, 16, 17]. Ectopic pregnancy is associated with psychological and physical pain among the affected persons [18, 19] and is potentially fatal and threatens the achievement of millennium development goal (MDG) number five on maternal mortality in Kenya [20, 21]. Various studies on the link between use of emergency contraception and ectopic pregnancy are inconclusive [1, 7, 9, 22-24]. Therefore, this study aimed to establish whether there is a significant association between LNG-EC emergency contraception and the occurrence of ectopic pregnancy. Understanding the relationship between incidences of ectopic pregnancies and emergency contraception is useful in designing sexual and reproductive health policy. This study can also act as a baseline for other related studies.

## Methods

This was a case control study on use of Levonorgestrel only emergency contraceptives among patients diagnosed with or without ectopic pregnancy at Moi Teaching and Referral Hospital (MTRH) in Eldoret-Kenya. The control group were clients with normal intrauterine pregnancy while the cases were women diagnosed with ectopic pregnancy. The study included a total of 316 participants; 79 cases and 237 controls in the ratio of 1:3 who were matched based on age, level of education, marital status and parity. All of the study participants were from Eldoret-town and its environs. They were interviewed for history of LNG-EC use as well as other risk factors for ectopic pregnancies. Associations between the outcome variable (ectopic pregnancy) and the exposure (LNG-EC use) was assessed using Pearson's Chi Square test. Fisher's exact test was used whenever the assumptions for use of Chi Square were violated. Univariate logistic regression model was used to assess the relationship between the outcome and each of the independent variables. The multivariate logistic regression model was used to assess the relationship between the outcome and the exposure (use of LNG-EC) adjusting for the confounding variables. The variables that were significantly associated with the outcome in the univariate analysis were included in the multivariate logistic regression model. Backward selection method was used to choose the variables to be retained in the model. The variables that had the greatest p-value greater than 0.05 were removed from the model in a stepwise manner. Family planning and use of LNG-EC, and history of treatment for STI and Depo Provera were associated hence family planning and history of treatment for STI were excluded from the model. Timing of use of LNG-EC following unprotected coitus was not included due to perfect correlation with use of LNG-EC. A logistic regression model was used to assess the relationship between the outcome and the exposure adjusting for confounders. This study was approved by the Institutional Research and Ethics Committee (IREC) of MTRH and Moi University School of Medicine and permission from MRTH obtained.

## Results

The mean age of the participants was 27.1 (± 5.4) years with a minimum and a maximum of 18.0 and 43.0 years respectively. Average menarche was 14.5 (±1.4) years with a minimum and a maximum of 11.0 and 19.0 years respectively (Table 1). The cases and the controls were similar by age (p = 0.990), education level (p = 0.850), marital status (p = 0.559), employment status (p = 0.186), spouse employment status (p = 0.483), and parity (p = 0.999). The timing of levonorgestrel-only emergency contraception (LNG-EC) use within the immediate cycle before the pregnancy was within 36 hours among all the study participants (Table 2); with majority (both cases and controls) taking their first dose between 13 to 24 hours. There was no evidence of association between the timing of LNG-EC use and occurrence of ectopic pregnancy (Table 2). History of family planning could not be retained in the model due to its association with use of LNG-EC. The use of LNG-EC pills adjusting for history of using depo Provera and menarche was associated with 9.4 times increased odds of developing ectopic pregnancy {OR: 9.34 (95% CI: 3.9 -16.0)} (Table 3).

**Table 1:** sociodemographic characteristics

	Mean ± SD or n (%)		
Variable	Cases, N = 79	Controls, N = 237	P-value
Age (Years)	27.1 ± 5.4	27.1 ± 5.4	0.990^t^
Education			
Primary/None	31 (39.2%)	90 (38.0%)	
Secondary	25 (31.6%)	70 (29.5%)	0.850^c^
College/University	23 (29.1%)	77 (32.5%)	
Marital status			
Single/Divorced	24 (30.4%)	62 (26.2%)	0.559^c^
Married	55 (69.6%)	175 (73.8%)	
Employment status			
Employed	15 (19.0%)	70 (29.5%)	
Unemployed	46 (58.2%)	119 (50.2%)	0.186^c^
Self-employed	18 (22.8%)	48 (20.3%)	

c Pearson’s Chi Square test

t Independent samples t-test

**Table 2:** comparison of gynecological characteristics between the cases and controls

Variable	Cases, N = 79	Controls, N = 237	P-value
Parity			
0	20 (25.3%)	62 (26.2%)	
1	30 (38.0%)	88 (37.1%)	
2	18 (22.8%)	54 (22.8%)	0.999^c^
3+	11 (13.95)	33 (13.9%)	
Menarche (Years)	14.8 ± 1.6	14.4 ± 1.3	0.023^c^
History of miscarriages	21 (26.6%)	57 (24.1%)	0.763^c^
History of family planning	46 (58.2%)	26 (11.0%)	<0.001^c^
Emergency contraceptive pills	26 (32.9%)	17 (7.2%)	<0.001^c^
Timing of LNG-EC use			
< 12 hours	6 (23.1) %	1 (5.9%)	
13-24 hours	17 (65.4%)	14 (82.4%)	0.320^f^
25-36 hours	3 (11.5%)	2 (11.8%)	
Depo Provera	14 (17.7%)	6 (2.5%)	<0.001^f^

c Pearson’s Chi Square test

f Fisher’s Exact test

**Table 3:** effect of LNG-EC pill on developing ectopic pregnancy adjusting for confounders

		Mean ± SD or n (%)			Univariate	Multivariate
Variable		Cases, N = 79	Controls, N = 237	P-value	OR (95% CI)	OR (95% CI)
LNG-EC		26 (32.9%)	17 (7.2%)	<0.001^c^	6.35 (3.21, 12.54)	9.34 (4.46, 19.56)
Depo Provera		14 (17.7%)	6 (2.5%)	<0.001^f^	8.29 (3.07, 22.43)	12.43 (4.37, 35.33)
Menarche (Years)		14.8 ± 1.6	14.4 ± 1.3	0.023^c^	3.17 (1.26, 7.97)	4.82 (1.67, 13.94)
History of family planning		46 (58.2%)	26 (11.0%)	<0.001^c^	11.31 (6.18, 20.71)	-
Timing of LNG-EC use	Never used	53 (67.1%)	220 (92.8%)			
	< 12 hours	6 (7.6)	1 (0.4%)		24.91(2.94, 11.29)	^-^
	13-24 hours	17 (21.5%)	14 (5.9%)	<0.001^f^	5.04 (2.34, 10.87)	-
	25-36 hours	3 (3.8%)	2 (0.8%)		6.23 (1.01, 38.20)	-
Age (Years)		27.1 ± 5.4	27.1 ± 5.4	0.990^t^	1.00 (0.95, 1.05	-
Education	Primary/None	31 (39.2%)	90 (38.0%)		Reference	
	Secondary	25 (31.6%)	70 (29.5%)	0.850^c^	1.04 (0.56, 1.91)	-
	College/University	23 (29.1%)	77 (32.5%)		0.87 (0.47, 1.61)	-
Marital status	Single/Divorced	24 (30.4%)	62 (26.2%)	0.559^c^	Reference	
	Married	55 (69.6%)	175 (73.8%)		0.81 (0.46, 1.42)	-
Employment status	Unemployed	46 (58.2%)	119 (50.2%)		Reference	
	Employed	15 (19.0%)	70 (29.5%)	0.186^c^	0.55 (0.29, 1.07)	-
	Self-employed	18 (22.8%)	48 (20.3%)		0.97 (0.51, 1.84)	-
Parity	0	20 (25.3%)	62 (26.2%)		Reference	
	1	30 (38.0%)	88 (37.1%)		1.06 (0.55, 2.03)	-
	2	18 (22.8%)	54 (22.8%)	0.999^c^	1.03 (0.50, 2.15)	-
	3+	11 (13.95)	33 (13.9%)		1.03 (0.44, 2.41)	-
History of miscarriages		21 (26.6%)	57 (24.1%)	0.763^c^	1.14 (0.64, 2.04)	-
History of treatment for STI		4 (5.1%)	2 (0.8%)	0.036^f^	6.27 (1.13, 34.90)	-

## Discussion

All methods of contraception can effectively reduce the number of intrauterine and ectopic pregnancies [25]. However, in the event of contraception failure, the risk of ectopic pregnancy is significantly increased in the women who become pregnant [26]. Previous studies have indicated that progesterone and its analogue Levonorgestrel, could inhibit human tubal activities which are considered as the main cause of impaired embryo-tubal retention and implantation [27]. Although the cases had similar experiences in history of miscarriages (26.6% vs. 24.1%, p = 0.763); they had a significantly higher proportion of use of family planning methods compared to the controls (58.2% vs. 11.0%, p < 0.001). They also had a significantly higher proportion of use of levonorgestrel-only emergency contraceptive pills compared to the controls (32.9% vs. 7.2%, p < 0.001). This significant risk of ectopic pregnancy following emergency contraception could be attributed to contraception failure as was noted in previous studies. When the use of LNG-EC was adjusted for history of use of depo Provera; it was associated with more than 9.34 times increased odds of developing ectopic pregnancy, OR: 9.34 (95% CI: 3.9 - 16.0). This study found a significant risk for the development of ectopic pregnancy among LNG-EC users compared to non-users. This could be attributed to a failure rate of 0.2-3.3% making it less effective in preventing pregnancy compared to other contraceptive methods such as oral contraceptives. Due to the easy accessibility to the Levonorgestrel only pills for emergency contraception, there is an increased uptake. However, there could be women who use it without strictly adhering to the guidelines [3, 18]. Studies done in china showed 5 and 4 times increased odds of developing ectopic pregnancy in those who had used Levonorgestrel emergency contraceptive pill compared to non-users respectively [12, 13]. Although the use of Levonorgestrel only pills for emergency contraception significantly influenced occurrence of ectopic pregnancy (p < 0.001) among the cases; the findings of this study clearly indicate that occurrence of ectopic pregnancy could also be influenced by the use of other contraceptives such as depo Provera (< 0.001). We established that menarche was associated with more than four times increased odds of occurrence of ectopic pregnancy. Other studies [28] have reported lack of such association, and further demonstrated that menarche is confounded by the age at coital debut. In our study we were unable to capture this data.

## Conclusion

One third of those who presented with ectopic pregnancy used levonorgestrel only emergency contraceptive pill. Levonorgestrel only emergency contraceptive pill use was associated with ectopic pregnancy. Women who use LNG-EC pill should be counselled on the increased risk of developing ectopic pregnancy. One of the limitations of this study is that we could not control for all confounders of ectopic pregnancy.

### What is known about this topic

- Levonorgestrel only emergency contraceptive pill is a well-established and recognized form of emergency contraception administered within 72 hours after of unprotected sexual intercourse;
- Levonorgestrel only emergency contraceptive pill is a risk factor in the occurrence of ectopic pregnancy;
- Previous studies on the link between emergency contraception use and ectopic pregnancy have not been conclusive.

### What this study adds

- Levonorgestrel only emergency contraceptive is associated with occurrence of ectopic pregnancy;
- Clients who had taken LNG-EC in the immediate cycle before the pregnancy had 9.34 times increased odds of developing ectopic pregnancy.

## Competing interests

The authors declare no competing interests.
